# The Mediterranean Species *Calendula officinalis* and *Foeniculum vulgare* as Valuable Source of Bioactive Compounds

**DOI:** 10.3390/molecules29153594

**Published:** 2024-07-30

**Authors:** Filomena Monica Vella, Domenico Pignone, Bruna Laratta

**Affiliations:** 1 National Research Council (CNR), Institute of Biosciences and BioResources (IBBR), Via P. Castellino 111, 80131 Naples, Italy; 2 Institute for Veterinary and Agri-Food Bioethics (IBV-A), 00054 Fiumicino, Italy; direttore@istitutoibva.it

**Keywords:** Mediterranean, *Calendula officinalis*, *Foeniculum vulgare*, phenols, terpenes, alkaloids, bioactivity

## Abstract

Research studies on plant secondary metabolites have increased over the last decades as a consequence of the growing consumer demand for natural products in pharmaceutics and therapeutics, as well as in perfumery and cosmetics. In this perspective, many Mediterranean plant species could be an appreciated source of bioactive compounds with pharmacological and health-promoting properties, including antioxidant, antimicrobial, antiviral, anti-inflammatory, and antitumor ones. *Calendula officinalis* and *Foeniculum vulgare* are commercially important plants of the Mediterranean flora, with great therapeutic use in the treatment of many disorders since ancient times, and are now listed in several world pharmacopoeias and drug agencies. The present review offers an overview of the main phytochemicals, phenols, terpenes, and alkaloids, biosynthesized in *C. officinalis* and *F. vulgare*, both species endemic to the Mediterranean region. Further, all current knowledge and scientific data on taxonomic classification, botanical description, traditional uses, pharmacological studies, and potential toxicity of both species were reported. The principal aim of this review is to point out the prospective use of *C. officinalis* and *F. vulgare* as valuable reservoirs of beneficial plant-derived products with interesting biological properties, also providing suggestions and future challenges for the full exploitation of these two Mediterranean species for human life improvement.

## 1. Secondary Metabolites, Biochemistry, and Biological Activity

The recent societal shift toward a sustainable lifestyle has resulted in increased demand for products derived from natural sources. Consequently, the consumption of plant secondary metabolites (SMs) is growing in interest for consumers and also for companies due to their promising biological activities, including antioxidant, antimicrobial, antiviral, and antitumor activities [1,2,3,4]. SMs have been found to have applications in many fields, such as supplements, nutraceuticals, pigments, cosmetics, bio-pesticides, herbicides, and bio-diesel due to their therapeutic and useful effects [3,4]. SMs, which are generally recognized as safe (GRAS), can be employed as alternatives to their synthetic counterparts, avoiding the undesirable toxic effects of chemicals on human wellness [2]. 

From an ecological point of view, unlike primary metabolites (such as sugars, amino acids, and nucleotides), SMs are not directly involved in essential plant functions like growth and reproduction though they play a crucial role in their long-term survival. Their role is multifaceted, encompassing plant communication, photo-protection, pest and parasite defense, and pollinator attraction [5]. Thus, the concentration of SMs varies seasonally and daily, and their production is influenced by numerous biotic and abiotic factors [1,4]. This literature review will demonstrate that these phytochemicals have a vast range of biological roles that are strictly determined by their chemical structures and are species-specific and organ-specific compounds [1,4]. 

The chemical classification typically divides SMs into three main groups depending on their biosynthetic pathways: phenols, terpenes, and alkaloids [1,4].

### 1.1. Phenols 

One of the largest and most complex biomolecules among phytochemicals is phenolic compounds. Based on the number of aromatic rings, carbon atoms, and hydroxyl groups, they are divided into different sub-classes: phenolic acids, flavonoids, and non-flavonoids compounds, the latter including stilbenes, lignans, and tannins [6,7]. Polyphenols are widely distributed in all plant organs. In particular, phenolic acids are generally found in seeds, leaves, roots, and stems, instead flavonoids are prominently in aerial parts, and tannins in roots, bark, and seeds [1]. 

Phenolic compounds act in plants mainly as defense and protective molecules against biotic and abiotic stresses [8]. To overcome the photo-oxidative stress, they act by maintaining the redox balance of plant cells and avoiding the generation of ROS (Reactive Oxygen Species) or quenching them. In addition, phenolic compounds can act as good UV screeners by absorbing the shortest solar wavelengths and reducing the highly energetic ones [9,10]. 

In detail, phenolic acids are divided into benzoic acid derivates (C_6_-C_1_; i.e., gallic acid, vanillic acid, syringic acid) and hydroxycinnamic derivates (C_6_-C_3_; i.e., caffeic acid, ferulic acid, coumaric acid) with promising therapeutic properties. In fact, for their antidepressants, anti-hypertensives, anti-inflammatory, neuroprotective, anti-hyperglycemic, anti-cancer, and antidiarrheal properties, they are considered versatile dietary components naturally present in all fruits and vegetables [11,12]. 

Flavonoids (C_6_-C_3_-C_6_) are mainly grouped into seven subclasses based on modifications to their basic skeletons: flavones (luteolin, apigenin), flavanols (hesperitin, naringenin, and eriodictyol), flavanones (naringin, hesperidin, eriocitrin), flavonols (quercetin, galangin, kaempferol, and myricetin), isoflavones (genistein, daidzein, and glycitein), and anthocyanins (cyanidin, delphinidin, malvidin) [13]. In plants, flavonoids are mainly found in the form of glycosides and are considered important molecules for the human diet, suggested as being active ingredients in food supplements and nutraceuticals, in the cosmetic field, and as natural dyes [13]. In fact, they are renowned for their several biological activities as antioxidant, anti-inflammatory, anti-cancer, and antihypertensive, and as circulation-improving and hypolipidemic agents [13].

Among the non-flavonoid compounds, tannins are classified as hydrolyzable (gallotannins and ellagitannins) and condensed (proanthocyanidins). They are mostly used in the veterinary field as anthelmintic and antimicrobial agents, as well as in the leather industry for their tanning properties [14].

### 1.2. Terpenes

Terpenes belong to the largest family of natural products. They are also known as isoprenoids since they originate from isoprene, a five-carbon atom compound, whose units are arranged in various structural patterns. Therefore, they are extremely diverse in structure, function, and properties, accounting for more than 50,000 known molecules. From a chemical point of view, they are classified according to the number of isoprene units into monoterpenes (C_5_H_8_)_2_, sesquiterpenes (C_5_H_8_)_3_, diterpenes (C_5_H_8_)_4_, sesterterpenes (C_5_H_8_)_5_, triterpenes (C_5_H_8_)_6_, tetraterpenes (C_5_H_8_)_8_, and so on [15,16]. Monoterpenes and sesquiterpenes are common components of essential oils and are responsible for the odorous properties of these compounds. Triterpenes are derived from the squalene biosynthetic pathway, through cyclization and various modifications to produce the diverse triterpene compounds. They contain numerous methyl groups that can be oxidized into alcohols, aldehydes, and acids, leading to various biologically active molecules. This group includes phytogenic bio-surfactants, historically utilized for their soap-like properties, and steroids, with cholesterol being the most significant representative. Finally, tetraterpenes are a class of terpenes composed of eight isoprene units, totaling 40 carbon atoms. They include carotenoids, like β-carotene, which are vital for photosynthesis and provide pigmentation in plants. Tetraterpenes exhibit antioxidant properties and serve as precursors for vitamin A synthesis [15,16]. 

In plants, the primary function of terpenes is to act as signaling molecules. Their emissions are linked to biotic and abiotic stresses, such as vital cycles, extreme temperature, radiation, drought, fire, air pollution, or herbivore attack [17]. The emission of monoterpenes and sesquiterpenes allows the plant to reduce ROS-induced damage and to improve ozone and thermal tolerance [18].

The growing interest in the potential application of terpenes can be attributed to their broad range of biological properties, including cancer chemoprevention, antimicrobial, antiviral, analgesic, anti-inflammatory, antifungal, and anti-parasitic activities [15,19]. Due to their numerous bioactivities, these SMs are demanded in several industrial sectors such as pharmaceuticals, food, cosmetics, perfumery, aromatherapy, and agricultural, and can be used as drugs, food supplements, flavors, fragrances, and bio-pesticides [1,20].

### 1.3. Alkaloids

Alkaloids are nitrogen-containing organic molecules that are very abundant in plants. So far, over 10,000 SMs have been classified as alkaloids from numerous families with a varied distribution in plant organs according to the phase of the life cycle [21].

Their name comes from the “alkali-like” nature of the nitrogen atoms present in their structure. These molecules include amine-type elements in the structure, usually in a heterocyclic ring. They are synthesized through various metabolic pathways, involving amino acids as precursors. In chemical classification, the alkaloids are categorized into three groups: true alkaloids, proto-alkaloids, and pseudo-alkaloids [22]. Specifically, true alkaloids have heterocyclic rings with nitrogen and are derived from amino acids; proto-alkaloids do not have heterocyclic rings with nitrogen and derive from amino acids; pseudo-alkaloids have heterocyclic rings with nitrogen and are derived from terpenoids or purines [22]. These SMs have wide-ranging biological activities, including analgesic, antimalarial, and stimulant properties, making them valuable in pharmacology and medicine. In plants, their biosynthesis is promoted as a consequence of abiotic and biotic stresses [23]. Therefore, they act as natural toxins for different organisms, defending plants from pathogens and preventing herbivore grazing. In apparent contrast, some alkaloids are fundamental for plant–pollinator interactions, thus favoring seed dispersion and plant reproduction [23]. For millennia, alkaloids have been used in all cultures as medicines, poisons, and drugs, and they are still important nowadays [22]. In fact, molecules such as stimulant alkaloids in coffee, tea, cacao, and nicotine in tobacco are consumed worldwide. Molecules with hallucinogenic, narcotic, or analgesic properties, such as morphine and atropine, have found applications in medicine [22]. Therefore, alkaloids are used as preparation for sedatives, stimulants, muscle relaxants, tranquilizers, and anesthetics, but also in therapy as antimalarial, antimicrobial, anti-diabetic, anti-cancer, anti-HIV, and antioxidants. Nevertheless, alkaloids are often abused being distributed as illegal drugs such as cocaine, heroin, and opium [22].

## 2. *Asteraceae* and *Apiaceae* Families

The families of *Asteraceae* and *Apiaceae* among Mediterranean plant species have been used since ancient times in folk medicine for the treatment of illnesses and pain relief [24,25,26,27]. They are also considered valuable reservoirs of botanical flavors and fragrances, utilized in foods and cosmetics as supplements and additives [24,25,26,27]. Today, these families have a cosmopolitan distribution and are easily adaptable around the world. 

The *Asteraceae* (*Compositae*) is the most abundant flowering plant family in many European countries: it consists of approximately 25,000 species and 1700 genera [24,27]. Demonstrating a high level of adaptability, species of this family are distributed worldwide, to different habitats and climatic conditions, except in Antarctica. This family includes a number of well-known food species, such as chicory, sunflower, and lettuce, as well as a number of medicinal plants, such as wormwood, chamomile, marigold, and dandelion [24,27].

Another major and popular family of flowering plants is the *Apiaceae* (*Umbelliferae*), which encompasses almost 400 genera and about 4000 species across the globe. The *Apiaceae* family mainly consists of aromatic plants, commonly used as food, spice, and ornamental plants, as well as for medical purposes, in perfumery, and in the pharmaceutical and cosmetic industries. The most economically important crops and herbs belonging to this family are celery, carrot, parsley, coriander, cumin, fennel, anise, and dill [25,26].

A phylogenetic relationship between *Asteraceae* and *Apiaceae* has been hypnotized based on some studies on phytochemicals, which identify molecules with similar structures [28,29]. In particular, several sesquiterpene lactones, based on skeletal stereo-structural types, have been surprisingly detected in *Asteraceae* species, since they are also representative of the *Apieceae* family [29]. In truth, both families are regarded as the richest plants for sesquiterpene lactones, such as germacranolides, guaianolides, eudesmanolides, eremophilanolides, and elemanolides [28]. All these sesquiterpenes have been used as medicines, poisons, flavorings, and fragrances for millennia [30]. The discovery that sesquiterpene lactones are the most important chemicals in allergies and systemic contact dermatitis was also intriguing [31]. The therapeutic properties of the guaianolide lactone group are well documented for the treatment of inflammation and cancer [30,32]. More recently, guaianolide lactones have been reported to be useful even in treating type-2 diabetes patients [33]. 

The basic skeletal types of guaianolide lactones of the *Apiaceae* and *Asteraceae* families are very similar. Despite the common structure of the γ-lactone function, they differ in the stereochemistry around the lactone ring. Lactone biosynthesis also has a similar pathway as is reported in both plant families, but the difference in the spatial arrangement and chemical configuration of some protons have been remarked on [29].

The genus *Calendula* is considered one of the largest and most evolved of the *Asteraceae* family [34]. This genus encompasses both annual and perennial plants, native to the Mediterranean basin [35,36]. The most common species are *Calendula officinalis* Linn., and *Calendula arvensis* Linn., with *C. officinalis* being the most studied species for medicinal purposes and its high economic value [37]. Nevertheless, a few studies have been carried out for the other species growing in the Mediterranean basin (*Calendula stellata*, *Calendula suffruticosa*, and *Calendula tripterocarpa*), as reported by Arora et al. [37]. Recently, seven other accepted species (*Calendula eckerleinii*, *Calendula karakalensis*, *Calendula lanzae*, *Calendula maroccana*, *Calendula meuselii*, *Calendula pachysperma*, and *Calendula palaestina*) have been reported [38]. This demonstrates the need for further investigation to understand the evolution of the genus *Calendula*.

An important member of the *Apiaceae* family is the Mediterranean endemic *Foeniculum vulgare* Mill. [39,40]. Today, fennel is the most studied culinary, medicinal, aromatic, and flavoring plant [39]. As reported by Malhotra [41], three main varieties have been described for *F*. *vulgare*: var. *piperitum* (bitter fennel), var. *dulce* (sweet fennel), and var. *azoricum* (Florence fennel or finocchio). Moreover, two subspecies of fennel are reported: *piperitum*, whose inflorescences and tops are used to make vinegar; and *capicellaceum*, which tastes bitter and whose seeds are still used to flavor liqueurs [42,43]. Nevertheless, there are only a few studies on the two subspecies.

## 3. Review Methods

For this review, the following international electronic databases were queried: Scopus, Web of Science, PubMed, Medline, and Google Scholar. Only original papers written in English were considered. Keywords used to search the databases included plant names (e.g., *Calendula*, *C. officinalis*, *Foeniculum*, *F. vulgare*) combined with names of each class of SMs considered (phenols, terpenes, alkaloids). Moreover, ethnobotanical knowledge, culinary uses, and biological activities associated with each species were searched to identify the main uses and potential future applications for both *C. officinalis* and *F. vulgare*.

To date, a large amount of scientific information on *Calendula* and *Foeniculum* species is available in the literature. Considering only *Calendula* species, a high number of studies were conducted during 2010–2019 (representing 44% of publications); furthermore, during 2020–2022, approximately 19% of studies on this genus were published, as reported by Olennikov and Kashchenko [38]. In the case of *Foeniculum* species, 20% of the manuscripts were published from 2001 to 2005. This value increased to approximately 38% from 2006 to 2010, until it reached 39% of the articles reported from 2011 to 2013, indicating the trend toward the scientific topic [40].

The main objective of this review is to better understand the prospective use of these two Mediterranean species as valuable sources of beneficial plant natural products with potential therapeutic properties, taking into account all the information available in the literature on the uses, phytochemicals, and pharmacological studies reported on *C. officinalis* and *F. vulgare*.

## 4. Calendula Officinalis

### 4.1. Taxonomic Classification and Botanical Description

*C. officinalis* belongs to the huge family of *Asteraceae*, as reported in Table 1 [44]. It is usually known as common marigold. The name Calendula derives from the Latin *calendae* means “the first day of the month”, referring to its blooming period [36].

**Figure 1:**
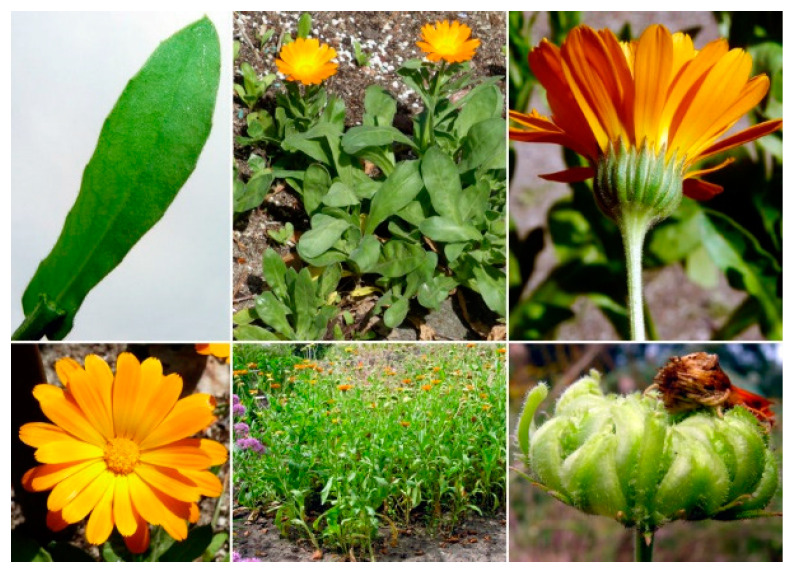
*C. officinalis* (https://dryades.units.it/floritaly/index.php, accessed on 22 June 2024).

*C. officinalis* is widely cultivated in sunny locations and usually grows in a variety of soils (acidic, sandy, and clayey). Although perennial, it is commonly treated as an annual or biennial plant. In temperate areas, seeds are sown in spring and typically bloom quickly in flowers [44]. It can reach a height of 30–60 cm with a stem angular, hairy, and solid (Figure 1). Lower leaves are spatulate, with a length of 10–20 cm long. The inflorescences comprise a thick capitulum or flower head of 4–7 cm diameter, surrounded by two rows of hairy bracts. In particular, flower heads vary in color from bright yellow to orange, and the corolla is around 15–25 mm long and 3 mm wide [37,44]. 

### 4.2. Ethnopharmacology and Human Food Uses

*C. officinalis* ranks among the oldest cultivated flowers, first described in the third century B.C.; ancient Romans and Greeks used calendula flowers in many rituals and ceremonies, to make crowns or garlands. The nickname marigold given to calendula derives from “Mary’s Gold”, referring to the use of the flowers in early Christian events [36]. The plant has been in cultivation and used for medicinal purposes only since the 12th century and has a long history of use. In the Middle Ages, calendula was used for hepatic disorders, poisoning, and cardiac tonicity. Doctors realized that the plant could stop bleeding and promote wound healing around the 18th century [36]. Traditionally, calendula has provided different uses, including the elaboration of food, dyes, cosmetics, and traditional remedies. Along with these uses, different plant parts of *C officinalis* have been used for medicinal purposes, above all leaves and flowers [35,37,45]. 

Currently, *C. officinalis* has been listed in multiple national pharmacopoeias and agencies, such as the European Pharmacopoeia, British Herbal Pharmacopoeia, and European Medicines Agency, for its well-known therapeutic applications such as antipyretic, anti-inflammatory, antiepileptic, and antimicrobial properties [46]. *C. officinalis* is used in wound healing and to treat internal inflammation, gastrointestinal ulcers, and dysmenorrhea, and as a diuretic and diaphoretic in convulsions [35,37,45]. Particularly, calendula tea is used as eyewash and to make gargles in the treatment of inflammatory conditions of the skin and mucous membranes [37]. Mother tincture of *C. officinalis* is involved in homoeopathy for the treatment of mental tension and insomnia [47].

Recently, calendula has assumed the function of an edible flower, improving the appearance, flavor, and aesthetic value of food, according to consumer’s tastes [35]. Other food uses of calendula petals include the addition of yellow color to foods as a substitute for saffron, as well as in decorations of cakes, sweets, and savory toppings. Fresh flowers are utilized in lettuce, rice, fish, herb butter, yogurt, and cheese spread, while the dried flowers are often used to make tea. The leaves, on the other hand, taste spicy and are well suited to flavorings soups and salads [48]. However, the use of this species in food products is subject to the removal of the pollen, as it may trigger severe allergic reactions [48]. Moreover, dyes and tinctures can be extracted from the dried and fresh flowers to produce a range of useful colors from yellow to orange [35].

### 4.3. Phytochemicals

Several classes of SMs have been reported in *C. officinalis* [37,38,49] and the main phytochemicals, phenols (phenolic acids, flavonoids), terpenes (monoterpenes, sesquiterpenes, saponins, carotenoids), and alkaloids are summarized in Table 2.

#### 4.3.1. Phenols

Phenolic acids and flavonoids have been identified in *C. officinalis* mainly from inflorescences, as shown in Table 2. Both benzoic acid and hydroxycinnamic acid derivates were reported among phenolic acids (Table 2). In particular, hydroxybenzoic acid, salicylic acid, protocatechuic acid, vanillic acid, syringic acid, hydroxycinnamic acid, ferulic acid, fumaric acid, chlorogenic acid, and caffeic acid were detected [50,51]. Considering flavonoids, Ak et al. [51] described the presence of hesperidin, catechin, miquelianin, isoquercitrin, rutin, cosmosiin, astragalin, nicotiflorin, quercetin, luteolin, and amentoflavone (Table 2). In addition, several flavonol 3-*O*-glycosides from *C. officinalis* flowers have been isolated and characterized by many authors, as reported in Table 2 [52,53,54,55]. Anthocyanins have been detected in calendula as the components of the flowers that tend to be red in color and are mainly glycosides of cyanidin, delphinidin, malvidin, paeonidin, pelargonidin, and petunidin [55].

#### 4.3.2. Terpenes

The terpenic profile of *C. officinalis* is mainly composed of monoterpenes, sesquiterpenes, triterpenes, and tetraterpenes (carotenoids), as shown in Table 2. 

Monoterpenes and sesquiterpenes are responsible for the odor of calendula flowers. The main monoterpenes isolated in *C. officinalis* are α-thujene, α-pinene, sabinene, β-pinene, limonene, 1,8-cineol, p-cymene, trans-β-ocimene, α-phellandrene, γ-terpenene, δ-3-carene, geraniol, bornyl acetate, and sabinyl acetate, as characterized by Okoh et al. [56] and Ak et al. [57]. Compounds of a sesquiterpene nature were detected in *C. officinalis* both in non-glycosidic and glycosides form. In particular, Okoh et al. [56] and Ak et al. [57] detected α-cubebene, α-copaene, α-bourbonene, β-cubebene, α-gurjunene, aromadendrene, β-caryophyllene, α-ylangene, α-humulene, epi-bicyclo-sequiphellandrene, germacrene D, allo-aromadendrene, β-salinene, calarene, α-muurolene, γ-muurolene, δ-cadinene, cadina-1,4-diene, α-cadinene, nerolidol, palustrol, β-oplopenone, α-cadinol, and τ-muurolol (Table 2). The sesquiterpene glycosides officinoside C and officinoside D are natural terpenes isolated exclusively from *C. officinalis*, in which a hydroxyl group is substituted by fucose, as characterized by Yoshikawa et al. [54].

Triterpenes in *C. officinalis* are present both in the free state and as an ester with fatty acids or alcohols, as well as in the glycosidic form, as listed in Table 2 [58,59,60,61,62,63]. Yoshikawa et al. [54] isolated four new triterpene glycosides, named calendasaponins A, B, C, and D (Table 2). Moreover, ten oleanane-type triterpene glycosides, including four new compounds, calendulaglycoside A 6′-*O*-methyl ester, calendulaglycoside A 6′-*O*-n-butyl ester, calendulaglycoside B 6′-*O*-n-butyl ester, and calendulaglycoside C 6′-*O*-n-butyl ester, were isolated from the flowers [52].

Approximately one hundred carotenoids (tetraterpenoids) in free and esterified forms have been found and identified in *C. officinalis* [64]. Owing to a wide range of petal colors, different types and amounts of carotenoids can be detected in calendula flowers. The carotenoids found in the petals were neoxanthin, 9Z-neoxanthin, violaxanthin, luteoxanthin, auroxanthin, 9Z-violaxanthin, flavoxanthin, mutatoxanthin, 9Z-anthroxanthin, lutein, 9/9A-lutein, 13/13Z-lutein, α-cryptoxanthin, β-cryptoxanthin, z-cryptoxanthin, lycopene, α-carotene, and β-carotene (Table 2). In addition, ten carotenoids were unique to orange-flowered cultivars. Among them, (5Z, 9Z)-lycopene, (5Z, 9Z, 5′Z, 9′Z)-lycopene, (5′Z)-γ-carotene, (5′Z, 9′Z)-rubixanthin, and (5Z, 9Z, 5′Z)-lycopene have been identified [65].

#### 4.3.3. Alkaloids

Few studies on the phytochemical characterization of *C. officinalis* describe the presence of alkaloid compounds (Table 2). Alkaloids, including sitsirikine, vinblastine, vindoline, catharanthine, and vinleurosine, have been identified in *C. officinalis* in detail [66] (Hernández-Saavedra et al., 2016). Moreover, in *C. officinalis,* significant quantities of pyrrolizidine alkaloids (platynecine-type) have been observed, with a share of 41.5% [34].

### 4.4. Biological Activities 

*C. officinalis* is registered as a herbal drug and several ailments have been treated with *C. officinalis* [36]. Actually, many scientific researches have established that *C. officinalis* has a wide spectrum of pharmacological effects, including having antioxidant, cardio-protective, antimicrobial, cytotoxic, anti-cancer, anti-diabetic, nootropic, anti-inflammatory, wound-healing, hepato-protective, nephro-protective, and antiviral properties [37,45], as itemized in Table 3.

Plant polyphenols such as phenolic acid and flavonoids are among the most significant natural compounds with biological properties. In particular, flavonoids of *C. officinalis* are involved in cardiovascular issues, as depicted in Table 3. Martinez [67] carried out a preclinical study to evaluate the effects of calendula flowers on the vascular smooth muscle of rats. A concentration-dependent relation was obtained in endothelium-denuded rat aortic rings, and the vaso-relaxant effect was attributed to the flavonoid quercetin. *C. officinalis* has also been proven to be cardio-protective against ischemic heart disease by stimulating left ventricular pressure and aortic flow, as well as by reducing myocardial infarct size and cardiomyocyte apoptosis [68]. In this research, cardio-protection appears to be achieved by modulating antioxidant and anti-inflammatory properties, but no information was provided on the calendula SMs involved.

*C. officinalis* flavonoids and phenolic acids showed strong radical-scavenging capacity and free radical protection. As reported by Rigane et al. [69], rutin, quercetin-3-*O*-glucoside, scopoletin-7-*O*-glucoside, isorhamnetin-3-*O*-glucoside, and gallic acid have been identified as major antioxidant phytoconstituents (Table 3). Moreover, petal and flower extracts tested for antioxidant activity by lipid peroxidation, indicated that the petal extract was more potent than the flower head [70]. Calendula exerts also anti-ROS and anti-reactive nitrogen species (RNS) activity in a concentration-dependent manner, with significant effects even at very low concentrations [71]. Moreover, Ak et al. [57] reported the sesquiterpene α-cadinol as the most abundant constituent of the essential oil with high antioxidant capacity through free radical scavenging and reducing mechanisms (Table 3).

The discovery and isolation of new bioactive compounds from medicinal plants is an immediate and pressing need due to the growing incidence of drug-resistant pathogens. With this in mind, the antimicrobial activity of calendula flowers against Gram-positive (*Escherichia coli* and *Staphylococcus aureus*), Gram-negative (*Salmonella typhae* and *Vibrio cholera*), and fungi (*Candida albicans*) was studied using different extraction solvents [47]. In particular, the ethanolic extract showed activity against *E. coli*, *V. cholera,* and *C. albicans*, whereas the methanolic extract was active only against *C. albicans*. The chloroform extract gave antimicrobial activity against all microbes, while the acetone extract was active only against *E. coli* [47]. However, the compounds or classes of molecules potentially involved in the mechanism of action linked to microbial growth inhibition were not identified in the study. The antimicrobial potential of methanol and ethanol extracts from *C. officinalis* petals was also evaluated against a panel of clinical microorganisms, including bacteria (*Bacillus subtilis*, *Pseudomonas aeruginosa*, *Bacillus cereus*, *E. coli*, *S. aureus*, *Klebsiella aerogenes*, *Enterococcus faecalis*, *Bacillus pumilis*, *Klebsiella pneumoniae*) and fungi (*C. albicans*, *Candida krusei*, *Candida glabrata*, *Candida parapsilosis*, *Aspergillus flavus*, *Aspergillus fumigatus*, *Aspergillus niger*, and *Exophiala dermatitidis*). Both extracts showed an antimicrobial activity comparable with the standard antibiotic, Fluconazole. Further clinical studies are required to examine the *C. officinalis* antimicrobial compounds [72]. In recent times, Darekar and Hate [73] investigated the antibacterial potential of chloroform extract of *C. officinalis* against *Bacillus subtilis*, *Klebsiella pneumonia*, *S. aureus*, and *Enterococcus faecalis*. The results revealed strong antibacterial activity against all tested strains. The study also aimed to identify phytochemicals with potential antibacterial activity present in *C. officinalis*. As a result, the major components of *C. officinalis* were caryophyllene (12.97%), lupeol (9.45%), stigmasterol (9.38%), and γ-sitosterol (5.07%), suggesting these terpenic biomolecules as potential calendula antibiotics (Table 3) [73]. 

*C. officinalis* has been shown to exhibit antimutagenic action. In particular, saponin-like triterpene compounds were employed in the screening of the antimutagenic activity, by using benzo-[a]pyrene, a well-known pro-mutagenic molecule [74]. *C. officinalis* flowers have shown in vitro cytotoxic activity, too. In particular, the triterpenic glycoside compounds, calendulaglycoside F6′-*O*-butyl ester and calendulaglycoside G6′-*O*-methyl ester (Table 3), resulted active against leukemia, colon cancer, and melanoma cell lines [52,75]. Recently, Cruceriu et al. [76] reported that *C. officinalis* could exert anti-cancer activity by inducing apoptosis, activating caspase 3 and caspase 7, and downregulating cyclin D1, D3, A, E, and several cyclin-dependent kinases, suggesting the prospective usage of *C. officinalis* in cancer management, particularly in cancer prevention, treatment, and palliative care for patients.

The triterpene saponins, calendasaponins A, B, C, and D, have shown a potent inhibitory effect on serum glucose levels. In fact, Yoshikawa et al. [54] demonstrated that *C. officinalis* flowers had a hypoglycemic effect, inhibitory activity of gastric emptying, and gastro-protective effect in glucose-loaded rats (Table 3). On the contrary, phenolic compounds in *C. officinalis* extracts were reported to exhibit weak inhibition against α-amylase and α-glucosidase, the main enzyme involved in decreasing postprandial hyperglycemia. In particular, the flower extract showed higher inhibition against α-amylase, followed by the leaf extract and the root extract, while the root extract was more active against α-glucosidase and flower one was the least active [51].

Nootropic activity was also reported in *C. officinalis*. Ercetin et al. [77] reported the enzyme inhibitory effects of *C. officinalis* extracts with different solvents (*n*-hexane, dichloromethane, acetone, ethyl acetate, methanol, and water) against acetylcholinesterase (AChE) and butyrylcholinesterase (BChE). The results revealed that the methanolic extracts of leaves and flowers have the highest activity against enzymes involved in cognitive metabolism, and therefore with potential to treat dementia and Alzheimer’s disease as nootropic agents (Table 3). 

Excellent anti-inflammatory activity was reported in *C. officinalis* (Table 3). Using in vivo pharmacological testing, it has been determined that the triterpenes fatty acid esters (lauryl, myristoyl, and palmitoyl esters of faradiol) are responsible for the anti-inflammatory effects of flower extract, as reported by Silva et al. [78]. They demonstrated that *C. officinalis* flower was much more effective for treating both acute and chronic swelling in mice. Further, the results showed that the potent anti-inflammatory response of *C. officinalis* extract may be mediated by the inhibition of pro-inflammatory cytokines (IL-6, IL-1β; TNF-α, and IFN-γ) and cyclooxygenase 2 (COX-2).

Calendula flowers may have an impact on the inflammatory process and the new tissue generation phase, as demonstrated by Nicolaus et al. [79], but the active compounds that are responsible are still a matter of debate. They found that while triterpenes may play a minor role, tetraterpenic compounds, such as carotenoids or their derivatives, may be more useful in the treatment of wound healing (Table 3). 

Further, tetraterpene extracts from *C. officinalis* flower are considered responsible for the protective role against hepato-toxicity and nephro-toxicity. Preethi and Kuttan [80] suggested that these activities are due to the presence of different carotenoids, such as lutein, zeaxanthin, and lycopene (Table 3).

Finally, the antiviral activity of *C. officinalis* flowers extract has been reported, as shown in Table 3. In particular, Bogdanova et al. [81] conducted a study on *Herpes simplex*, Influenza A2, and Influenza APR8 in vitro, and found that *C. officinalis* flowers extract was an effective agent against these viruses. Afterward, Kalvatchev et al. [82] demonstrated that *C. officinalis* flowers exhibited potent anti-HIV activity. This property was attributed to the inhibition of human immunodeficiency virus type 1 (HIV-1) reverse transcriptase in a dose-dependent manner as well as to the suppression of the replication of HIV-1. Until today, no studies have been conducted in order to identify the phytochemicals of *C. officinalis* involved in the antiviral mechanism.

### 4.5. Safety

Calendula cream and products have shown very few allergic and side effects, approximately 2% of the patients have reacted to skin contact with calendula products [83]. Generally, in the *Asteraceae* family, the only main group of chemicals that may cause allergies and contact dermatitis is sesquiterpene lactones [31,84]. 

## 5. Foeniculum Vulgare 

### 5.1. Taxonomic Classification and Botanical Description

The genus *F. vulgare* is a member of the family *Apiaceae* and is classified as shown in Table 4 [40]. The plant was placed in genus *Anethum* by Linnaeus, but later placed in the new genus *Foeniculum* by De Candolle [41]. The name *Foeniculum*, used by the Romans, is diminutive of the Latin *foenum*, meaning hay, given that fennel smells like hay.

**Figure 2:**
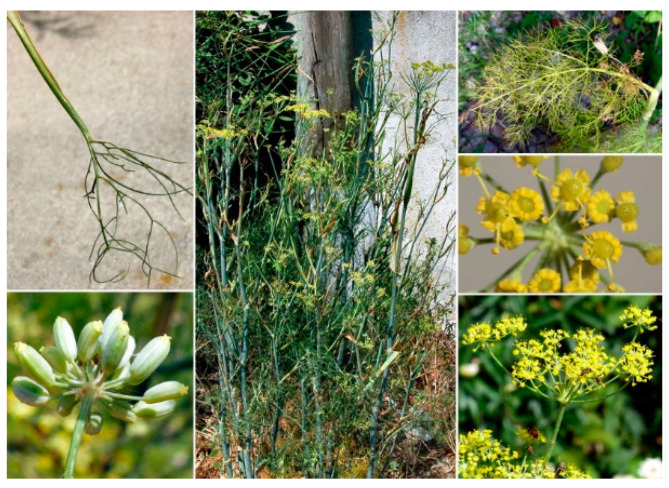
*F. vulgare* (https://dryades.units.it/floritaly/index.php, accessed on 22 June 2024).

Fennel is an herbaceous and aromatic plant comprising biennial or perennial varieties [40]. *F. vulgare* is commonly cultivated in tropical and temperate regions and this herbaceous plant is grown in semi-arid or arid environments [40]. In the Italian regions, especially in the south, in stony and sub-mountains up to an altitude of 700 m and along the coasts, wild spontaneous species of *F. vulgare* are still present with perennial plants, provided with robust and fittoning roots that form a false bulb named grumolo [43]. Fennel is characterized by stems grooved and intermittent leaves. Flowers are usually bisexual with yellow umbrellas in the form of oval beads (Figure 2). Fennel diachenes have a narrow, long, and cylindrical appearance with a length of about 8 mm and a width of 3 mm, with an aromatic odor and sweet taste [41].

Over the past few decades, modern horticultural practices have favored the use of seeds derived from careful germplasm selection and/or new varieties selected through genetic improvement programs, in contrast with the old practice of self-production of seeds that had brought to notice some important fennel landraces, particularly adapted to specific regional environments. Nevertheless, it is still possible to find varieties such as the Dolce di Firenze, Nostrale di Chioggia, Romanesco, Marchigiano, Mantovano, Di Bologna, and Di Napoli, which derive from the careful selection of germplasm and fixation of desired characters [43]. 

### 5.2. Ethnopharmacology and Human Food Uses

*F. vulgare* was renowned by the ancient Egyptians, Romans, Indians, and Chinese. In early Sanskrit writings, fennel was known as *madhurika* and its cultivation in India dates back to at least 2000 BC. To the ancient Greeks, fennel represented success and was called *marathon* because the battle of Marathon (490 BC) was fought in a fennel field [40]. Fennel was also a triumph symbol for the Romans and leaves were used to crown winners of games. Emperor Charlemagne encouraged the cultivation of fennel in Central Europe for its therapeutic properties [40]. Chewing the diachenes was believed to be important in curing stomach indisposition in the Middle Ages. In the 5th century, fennel was thought to have a sedative effect and, from the 9th century, numerous therapeutic properties were attributed to this plant [85]. 

*F. vulgare* is widely used in traditional medicine for a number of conditions and is recognized as an alternative medicine in various traditional systems of medicine like the Ayurveda, Unani, Siddha, Indian, and Iranian [40]. Different parts of the plant are employed to treat many digestive ailments [85]. It also is very useful in the treatment of diabetes, bronchitis, chronic cough, and kidney stones [39,40,85]. Due to its diuretic effect, fennel is also used to treat kidney and bladder diseases, and to relieve nausea. Further, it is applied to improve eye illnesses such as cataracts and conjunctivitis [85]. 

As it is a highly aromatic and flavorful herb, fennel is traditionally employed for culinary purposes. Fennel was considered a royal spice, served to kings with fruit, bread, and in fish dishes as early as the 13th century [41]. Today, all parts of the fennel plant are edible: diachenes, leaves, stalks, and false bulbs are regularly consumed in modern French and Italian cooking. Flowers and leaves are also utilized to make yellow and brown dyes [41]. Fennel diachenes are anise-like in aroma and are used as flavorings in baked products, meat and fish dishes, ice cream, alcoholic beverages, and herb mixtures [86]. The false bulb is a crisp vegetable and may be sautéed, fried, stewed, braised, grilled, or eaten raw [39].

### 5.3. Phytochemicals

Fennel, one of the most appreciated sweet and aromatic greens, raw or cooked, has a low energy content but is particularly rich in beneficial substances. Research led to the isolation and characterization of phytochemicals from *F. vulgare*, including phenolic acids, flavonoids, stilbenes, terpenes, and alkaloids (Table 5). 

#### 5.3.1. Phenols

Fennel fruits and diachenes are characterized to be rich in phenolic compounds, in particular phenolic acids and flavonoids, as shown in Table 5. Especially, *F. vulgare* fruits have been reported to contain 3-*O*-caffeoylquinic acid, 4-*O*-caffeoylquinic acid, 5-*O*-caffeoylquinic acid, 1,3-*O*-di-caffeoylquinic acid, 1,4-*O*-di-caffeoylquinic acid, 1,5-*O*-di-caffeoylquinic acid, as phenolic acids [87]. The flavonoids like eriodictyol-7-rutinoside and quercetin-3-rutinoside have also been isolated from *F. vulgare* fruit [87]. *F. vulgare* diachenes were reported to contain rosmarinic and chlorogenic acids as major phenolic acids (14.9% and 6.8%), and quercetin and apigenin as major flavonoids (17.1% and 12.5%), as demonstrated by Roby et al. [88]. As listed in Table 5, flavonoids quercetin-3-*O*-galactoside, kaempferol-3-*O*-rutinoside, kaempferol-3-*O*-glucoside, quercitin-3-*O*-glucuronide, kaempferol-3-*O*-glucuronide, isoquercitin and isorhamnetin-3-*O*-glucoside have also been reported to occur in *F. vulgare* [89]. 

Two new phenolic compounds, identified as diglucoside stilbene trimers (named Foeniculoside X and Foeniculoside XI) have also been isolated from *F. vulgare* fruits together with cis-miyabenol C, trans-miyabenol C, trans-resveratrol-3-*O*-β-D-glucopyranoside, sinapyl glucoside, syringin-4-*O*-β-glucoside, oleanolic acid, 7a-hydroxycampesterol, (3b,5a,8a,22E) 5,8-epidioxy-ergosta-6,22-dien-3-ol, and 2,3-dihydropropylheptadec-5-onoate, as reported in Table 5 [90].

#### 5.3.2. Terpenes

The characteristic anise odor of *F. vulgare* is mainly due to the monoterpenes and sesquiterpenes that mainly constitute this essential oil. Fennel has been reported to contain about 80 different monoterpenic compounds, the major ones being trans-anethole, fenchone, estragole (methyl-chavicol), p-anisaldehyde, and α-phellandrene, nerol, α-pinene, γ-terpinene, o-cymene, D-limonene, and β-myrcene, as shown in Table 5 [91,92,93,94,95,96,97,98]. The relative concentration of these compounds varies considerably depending on the phenological phase and geographical origin of the plant [91]. Further, the terpenic composition of *F. vulgare* exhibits considerable chemo-diversity depending upon the method of extraction and the accumulation of these compounds is different in each fennel part (roots, stem, diachenes, flowers, and fruits), as reported by Diaz-Maroto et al. [91]. 

Sesquiterpenes compounds present in *F. vulgare* are listed in Table 5. In particular, caryophyllene, germacrene D, bergamotene, β-farnesene, α-farnesene, α-curcumene were identified [99,100].

#### 5.3.3. Alkaloids

Fennel fruits were reported to contain alkaloids. In fact, Kaur and Arora [101] performed qualitative and quantitative phytochemical analyses on *F. vulgare* diachenes, demonstrating the presence of 2.80% alkaloids. Moreover, the presence of pyrrolizidine alkaloids was reported in *F. vulgare*, as depicted in Table 5 [102,103]. This large group of SMs was reported to be responsible for multiple cases of food and feed poisoning over the last 100 years [100,101]. 

### 5.4. Biological Activities

*F. vulgare* is officially noted in different national pharmacopoeias as an important part of polyherbal formulations in the treatment of many diseases and disorders like abdominal pains, arthritis, conjunctivitis, constipation, diarrhea, fever, gastritis, insomnia, irritable colon, mouth ulcer, stomach-ache, respiratory disorders, skin diseases, and so on [40]. Several pharmacological studies have reported that *F. vulgare* has an important variety of biological activities, comprising antioxidant, antimicrobial, antiviral anti-inflammatory, anti-cancer, hepato-protective, cardio-protective, gastro-protective, anti-cholesterol, anti-diabetic, estrogenic, anti-anxiety, and nootropic properties, as summarized in Table 6 [40,85].

*F. vulgare* is known as an excellent source of natural antioxidants. Fennel extracts can inhibit free radicals due to their high content of phenolic acids and flavonoids, such as caffeoylquinic acid derivates, rosmarinic acid, eriodictyol-7-rutinoside, quercetin-3-*O*-galactoside, and kaempferol-3-*O*-glucoside [87,88]. Fennel essential oil was also reported to possess antioxidant activity associated with the monoterpene components [93,94,98], as itemized in Table 6. 

Fennel is used to treat many bacterial, fungal, and viral infectious diseases. In particular, *F. vulgare* is characterized by antimicrobial effects on human pathogens and foodborne microorganisms. Among human pathogenic bacteria, Zellagui et al. [104] carried out the antimicrobial assay against Gram-positive (*Staphylococcus epidermidis*, *Staphylococcus saprophyticus*, *Staphylococcus blanc*) and Gram-negative bacteria (*E. coli*, *Proteus mirabilis*, *Proteus vulgaris*), and three fungal strains (*Aspergillus versicolor*, *Aspergillus fumigates* and *Penicillium camemberti*). Seven oxygenated monoterpenes, isolated and characterized from the aerial parts of *F. vulgare*, were tested and all microorganisms were inhibited [104]. The authors suggested that the antimicrobial activity of *F. vulgare* extracts can be attributed to the content of oxygenated monoterpenes by means of a mechanism that involves membrane disruption. Considering foodborne pathogens, Dadalioglu and Evrendilek [105] studied the chemical compositions and inhibitory effects of fennel essential oil on *E. coli*, *Listeria monocytogenes*, *Salmonella typhimurium*, and *S. aureus*. The results showed that the inhibitory effects of *F. vulgare* may be attributed to the main compound, trans-anethole (Table 6). These outcomes were also confirmed by Cetin et al. [106] who determined the chemical compositions of the essential oil from the inflorescence, leaf stems, and aerial parts of fennel, and their antimicrobial activities. The study revealed that trans-anethole, the main component, is responsible for the antimicrobial activity (Table 6). 

Orhan et al. [107] studied the antiviral activity of the fennel essential oil against the DNA virus Herpes simplex type-1 (HSV-1) and the RNA virus parainfluenza type-3 (PI-3), recording a significant inhibition from *F. vulgare*. Moreover, trans-anethole was tested and was reported as the main compound for the antiviral activity of fennel (Table 6).

Monoterpenes present in *F. vulgare* are considered to be associated with the prevention of several disorders induced by oxidative stress, such as cardiovascular disease, cancer, and inflammation. In particular, Chainy et al. [108] showed that trans-anethole is responsible for the suppression of both inflammation and carcinogenesis (Table 6). This bioactive compound was reported to act at an early step in the cascade of TNF-dependent signal transduction, so inhibiting cytokine-induced cellular response was associated with both diseases. The in vitro cyto-protection activity of *F. vulgare* was also estimated against normal human blood lymphocytes and the B16F10 melanoma cell line [109]. These results suggest that fennel could be considered a natural source of antitumor agents as well as being cyto-protective to normal cells. Moreover, fennel was proven to have significant anti-cancer activity against breast cancer cells (MCF-7) and liver cancer (HepG), as reported by Mohamad et al. [110]; nevertheless, no information was provided about the phytochemicals of *F. vulgare,* presumably involved in the anti-cancer mechanism.

One of the most common uses of *F. vulgare* has been to lower blood pressure by causing diuresis and increasing the excretion of sodium and water from the body. Significant antithrombotic activity and inhibition of platelet aggregation were observed in mice after oral administration of fennel essential oil and its most abundant phytochemical trans-anethole [95]. 

In a study conducted by Ozbek et al. [111], the hepatotoxicity caused by CCl4 administration in rats was inhibited by *F. vulgare* essential oil. In this research, the decreased levels of serum aspartate aminotransferase (AST), alanine aminotransferase (ALT), alkaline phosphatase (ALP), and bilirubin were demonstrated, and D-limonene and β-myrcene were suggested to be the components responsible for the potent hepato-protective action (Table 6).

It has been shown that fennel has a positive effect on gastrointestinal disorders. In fact, *F. vulgare* plays a protective role against ethanol-induced gastric mucosal lesions, as a consequence of a reduction in lipid peroxidation and augmentation in the antioxidant activity, as reported by Birdane et al. [112]. Moreover, Al-Mofleh et al. [113] also demonstrated the protective effect of fennel on gastric ulcers. In both papers, it was proposed that this property was linked to the antioxidant capacity of fennel, but no investigation was carried out to exactly identify the phytochemicals involved in the mechanism. Instead, Tognolini et al. [95] tested trans-anethole in rats with ethanol-induced gastric lesions and demonstrated that this compound plays the role of a gastro-protecting molecule (Table 6). 

The study of the anti-cholesterol and anti-atherogenic effect of methanolic extract from *F. vulgare* showed that the treatment significantly reduced plasma lipid levels, facilitating blood flow in the coronary and preventing fatty deposits in the arteries [114]. Further, fennel extracts were demonstrated to be useful for the control of blood glucose in diabetic patients. In fact, daily use of the extract could be effective in reducing chronic complications associated with diabetes [115]. *F. vulgare* was also reported to reduce blood glucose and triglycerides and, contemporarily, increase levels of liver and muscle glycogen [116]. Consequently, *F. vulgare* can be used in the pharmaceutical industry for the manufacture of anti-diabetic drugs [117], but further investigation is needed to understand the mechanism of action.

Fennel has been used for thousands of years as an estrogenic agent. As a consequence of this property, fennel increases milk secretion, reduces menstrual pain, facilitates birth, and increases sexual desire. Trans-anethole is the main estrogenic molecule in extract and essential oil from fennel, being the methyl ether of estrone [118]. Different quantities of fennel significantly decreased contraction intensity induced by oxytocin and prostaglandins, as showed by Ostad et al. [119]. On the other hand, Myrseyed et al. [120] demonstrated the effect of fennel extracts in reducing testosterone, FSH, and LH levels and sperm amount, thus suggesting a negative effect on male reproductive activity.

Fennel is also a drug used for the treatment of anxiety and stress. It relieves psychological and physical symptoms associated with these conditions. Mesfin et al. [121] evaluated the use of *F. vulgare* essential oil in stress and anxiety management in a mice model. They demonstrated that the group treated with fennel essential oil had much lower agitation and stress levels than the control group. The calming properties of fennel may be linked to phytoestrogens and to trans-anethole (methyl ether of estrone), which are involved in the phenomenon of anxiety mediated by the GABA-ergic system and estrogen receptors [122]. In another study [123], limonene, a minor component of the *F. vulgare* essential oil, has also been reported to have anxiety-relieving properties (Table 6).

Nootropic activity was also reported in *F. vulgare*. In fact, there is some evidence in favor of the use of *F. vulgare* for the treatment of cognitive disorders like dementia and Alzheimer’s disease. Joshi and Parle [124] administered *F. vulgare* for eight successive days to mice. They registered an amelioration in the amnesic effect of scopolamine and in the aging-induced memory deficits, concluding that fennel may be employed in the treatment of cognitive disorders (dementia and Alzheimer’s disease) as a nootropic and anticholinesterase agent. However, no information was provided on the fennel phytoconstituents involved.

### 5.5. Safety

Extracts and essential oils of fennel can be considered safe due to their long history of ethnomedicinal use with no reports of serious adverse effects. However, estragole (methyl-chavicol) has become a concern in recent years because of its structural similarity to methyl-eugenol present in *F. vulgare*. This has led the European Union (EU) to issue a new legal limit for estragole of 10 mg/kg in non-alcoholic beverages [125]. Further, the Scientific Committee on Food (SCF) of the European Union restricts the use of this substance.

The ability of estragole to cause genotoxicity and, thus, to be carcinogenic was first described by Drinkwater et al. [126] and then followed by numerous in vivo and in vitro studies [127,128,129,130,131]. To the present date, the potential of estragole to induce carcinogenesis in humans remains unclear. The critical factor for estragole’s carcinogenicity is its metabolic activation, leading to the formation of unstable molecules that form adducts with nucleic acids, damaging DNA. Estragole metabolism is dose-dependent and elevated doses of estragole increase its biotransformation, leading to the formation of mutagenic metabolites [132]. 

## 6. Challenges and Future Perspectives

The Mediterranean is one of the most biologically diverse regions on the planet. It was recently declared an “Intangible Cultural Heritage of Humanity” by UNESCO for its rich cultures, customs, beliefs, environment, and diet. With 25,000 plant species, 13,000 of which are endemic, it is the third richest area in the world in terms of plant species and is considered one of the world’s biodiversity hotspots. However, global climate change may pose a serious environmental threat to the region due to increased drought periods and heat waves. In order to survive in these worrying climates, plants have evolved various mechanisms, including the synthesis of an extraordinary array of secondary metabolites, which act mainly as plant defense compounds against environmental stress. 

This paper reviewed the literature on the main SMs, phenols (phenolic acids, flavonoids), terpenes (monoterpenes, sesquiterpenes, saponins, carotenoids), and alkaloids, biosynthesized in Mediterranean *C. officinalis* and *F. vulgare.* Until the present, many papers have been published on bioactive compounds in calendula and fennel, but only a few of them also reported the biochemical/ecological aspect, and none of them in recent times. In fact, as SMs are strongly influenced by the genotype–environment interaction, it would be interesting to encourage the study of environmental features that maximize the production of these valuable biomolecules and to study how the climatic changes can modify the amounts and the type of SMs biosynthesized by these two Mediterranean species. 

Nowadays, *C. officinalis* and *F. vulgare* are considered treasured sources of phenols, terpenes, and alkaloid compounds, with a wide array of therapeutic, pharmacological, and health-promoting properties. Particularly, antioxidant, antimicrobial, antiviral, anti-inflammatory, anti-cancer, anti-diabetic, cardio-protective, hepato-protective, nootropic, and skin-protective activities are the main biological properties reported for both species. Moreover, *C. officinalis* showed wound-healing and nephro-protective features, while *F. vulgare* exhibited estrogenic and anti-anxiety attributes. 

One of the main outcomes of the study shows that among SMs with interesting biological activities, the sesquiterpene lactones, biomolecules that have been abundantly used as medicine, poison, flavoring, and fragrance for several millennia, are abundant in these two botanical families *Asteraceae* and *Apiaceae.* However, no studies have been carried out on *C. officinalis* and *F. vulgare* and further efforts are needed to identify and characterize these bioactive compounds in calendula and fennel.

It is interesting to notice that the use of only one plant part (flowers for *C. officinalis* and diachenes for *F. vulgare*) is the rule, while the rest of the biomass (leaves, stems, roots) is considered a waste that is typically unexploited and understudied. Hence, it is crucial that extensive research on all plant parts of calendula and fennel is conducted in the future. Plant wastes and by-products are high-added resources to obtain appreciated natural products, fully respecting the transition from a linear to a circular management present in the objectives of the European Union’s Circular Economy Action Plan. 

## Figures and Tables

**Table 1:** Taxonomic classification of *C. officinalis* [44].

Rank	Scientific Name
Kingdom	*Plantae*
Division	*Magnoliophyta*
Class	*Magnoliopsida*
Order	*Asterales*
Family	*Asteraceae*
Genus	*Calendula*
Species	*C. officinalis*

**Table 2:** Main chemical compounds in *C. officinalis*.

Compounds	Plant Part	References
**Phenols**		
*Phenolic acids*		
Hydroxybenzoic acid, salicylic acid, protocatechuic acid, vanillic acid, syringic acid, hydroxycinnamic acid, ferulic acid, fumaric acid, chlorogenic acid, caffeic acid	flowers	[50]
	flowers, leaves, roots	[51]
*Flavonoids*		
Hesperidin, catechin, miquelianin, isoquercitrin, rutin, cosmosiin, astragalin, nicotiflorin, quercetin, luteolin, amentoflavone	flowers, leaves, roots	[51]
Isorhamnetin 3-*O*-neohesperidoside, isorhamnetin 3-*O*-rhamnosylrutinoside, isorhamnetin 3-*O*-rutinoside, isorhamnetin 3-*O*-glucoside, isorhamnetin-3-*O*-(6″-acetyl)-glucoside, quercetin-3-*O*-rhamnosylrutinoside, quercetin-*O*-pentosylhexoside, quercetin 3-*O*-glucoside, quercetin 3-*O*-rutinoside, quercetin-*O*-acetyldeoxyhexosylhexoside, quercetin-3-*O*-(6″-acetyl)-glucoside, rutinoside, neohesperidoside, quercetin glucoside, kaempferol-*O*-rhamnosylrutinoside	flowers	[52,53,54,55]
Cyanidin, delphinidin, malvidin, paeonidin, pelargonidin, petunidin (glycosides)	flowers	[55]
**Terpenes**		
*Monoterpenes*		
α-thujene, α-pinene, sabinene, β-pinene, limonene, 1,8-cineol, p-cymene, trans-β-ocimene, α-phellandrene, γ-terpenene, δ-3-carene, geraniol, bornyl acetate, sabinyl acetate	leaves, flowers	[56,57]
*Sesquiterpenes*		
α-cubebene, α-copaene, α-bourbonene, β-cubebene, α-gurjunene, aromadendrene, β-caryophyllene, α-ylangene, α-humulene, epi-bicyclo-sequiphellandrene, germacrene D, allo-aromadendrene, β-salinene, calarene, α-muurolene, γ-muurolene, δ-cadinene, cadina-1,4-diene, α-cadinene, nerolidol, palustrol, β-oplopenone, α-cadinol, τ-muurolol	leaves, flowers	[56,57]
Officinoside C, officinoside D	flowers	[54]
*Triterpenes*		
Sitosterol, stigmasterol	seedlings, leaves	[58]
3-monoesters taraxasterol, lupeol	flowers	[59,60]
Ursadiol	flowers	[61,62]
Faradiol-3-*O*-palmitate, faradiol-3-*O*-myristate, faradiol-3-*O*-laurate, arnidiol-3-*O*-palmitate, arnidiol-3-*O*-myristate, arnidiol-3-*O*-laurate, calenduladiol-3-*O*-palmitate, calenduladiol-3-*O*-myristate	flowers	[60,63]
Calendasaponins A, B, C, and D, officinosides A, and B	flowers	[54]
Calendulaglycoside A, calendulaglycoside A 6′-*O*-methyl ester, calendulaglycoside A 6′-*O*-n-butyl ester, calendulaglycoside B, calendulaglycoside B 6′-*O*-n-butyl ester, calendulaglycoside C, calendulaglycoside C 6′-*O*-methyl ester, calendulaglycoside C 6′-*O*-n-butyl ester, calendulaglycoside F 6′-*O*-butyl ester, calendulaglycoside G 6′-*O*-methyl ester	flowers	[52]
*Carotenoids*		
Neoxanthin, 9Z-neoxanthin, violaxanthin, luteoxanthin, auroxanthin, 9Z-violaxanthin, flavoxanthin, mutatoxanthin, 9Z-anthroxanthin, lutein, 9/9A-lutein, 13/13Z-lutein, α-cryptoxanthin, β-cryptoxanthin, z-cryptoxanthin, lycopene, α-carotene, β-carotene	flowers	[64]
(5Z, 9Z)-lycopene, (5Z, 9Z, 5′Z, 9′Z)-lycopene, (5′Z)-γ-carotene, (5′Z, 9′Z)-rubixanthin, (5Z, 9Z, 5′Z)-lycopene	flowers	[65]
**Alkaloids**		
Sitsirikine, vinblastine, vindoline, catharanthine, vinleurosine	flowers	[66]
Platynecine, platynecine-type	aerial parts	[34]

**Table 3:** Pharmacological activities along with their phytochemical constituents in *C. officinalis*.

Pharmacological Activity	Phytochemicals	References
Cardio-protective	Phenols	[67]
	-	[68]
Antioxidant	Phenols	[69]
	-	[70]
	-	[71]
	Sesquiterpenes	[57]
Antimicrobial	-	[47]
	-	[72]
	Sesquiterpenes	[73]
Cytotoxic and anti-cancer	Triterpenes	[74]
	Triterpenes	[75]
	Triterpenes	[52]
	-	[76]
Anti-diabetic and hypoglycemic	Phenols	[51]
	Triterpenes	[54]
Nootropic	Triterpenes	[77]
Anti-inflammatory	Triterpenes	[78]
Wound-healing	Tetraterpenes	[79]
Hepato-protective and nephro-protective	Tetraterpenes	[80]
Antiviral	-	[81]
	-	[82]

**Table 4:** Taxonomic classification of *F. vulgare* [40].

Rank	Scientific Name
Kingdom	*Plantae*
Division	*Tracheophyta*
Class	*Magnoliopsida*
Order	*Apiales*
Family	*Apiaceae*
Genus	*Foeniculum*
Specie	*F. vulgare*

**Table 5:** Main chemical compounds in *F. vulgare*.

Compounds	Plant Part	References
**Phenols**		
*Phenolic acids*		
3-*O*-caffeoylquinic acid, 4-*O*-caffeoylquinic acid, 5-*O*-caffeoylquinic acid, 1,3-*O*-di-caffeoylquinic acid, 1,4-*O*-di-caffeoylquinic acid, 1,5-*O*-di-caffeoylquinic acid	fruits	[87]
Rosmarinic acid, chlorogenic acid	diachenes	[88]
*Flavonoids*		
Eriodictyol-7-rutinoside, quercetin-3-rutinoside	fruits	[87]
Quercetin, apigenin	diachenes	[88]
Quercetin-3-*O*-galactoside, kaempferol-3-*O*-rutinoside, kaempferol-3-*O*-glucoside, quercitin-3-*O*-glucuronide, kaempferol-3-*O*-glucuronide, isoquercitin, isorhamnetin-3-*O*-glucoside	whole plants	[89]
*Stilbenes*		
Foeniculoside X, Foeniculoside XI, cis-miyabenol C, trans-miyabenol C, trans-resveratrol-3-*O*-β-D-glucopyranoside, sinapoyl glucoside, syringin-4-*O*-β-glucoside, oleanolic acid, 7a-hydroxycampesterol, (3b,5a,8a,22E) 5,8-epidioxy-ergosta-6,22-dien-3-ol, 2,3-dihydropropylheptadec-5-onoate	fruits	[90]
**Terpenes**		
*Monoterpenes*		
Trans-anethole, estragole, fenchone, p-anisaldehyde, α-phellandrene, nerol, α-pinene, γ-terpinene, o-cymene, D-limonene, β-myrcene	stems	[91]
	diachenes	[92,93]
	whole plants	[94,95]
	leaves, diachenes	[96,97]
	fruits	[98]
*Sesquiterpenes*		
Caryophyllene, germacrene D	aerial parts	[99]
Bergamotene, β-farnesene, α-farnesene, α-curcumene	fruits	[100]
**Alkaloids**		
Pyrrolizidine alkaloids	fruits	[101]
	leaves	[102,103]

**Table 6:** Pharmacological activities along with their phytochemical constituents in *F. vulgare*.

Pharmacological Activity	Phytochemicals	References
Antioxidant	Phenols	[87]
	Phenols	[88]
	Monoterpenes	[98]
	Monoterpenes	[93]
	Monoterpenes	[94]
Antimicrobial	Monoterpenes	[104]
	Monoterpenes	[105]
	Monoterpenes	[106]
Antiviral	Monoterpenes	[107]
Anti-inflammatory and anti-cancer	Monoterpenes	[108]
	-	[109]
	-	[110]
Hepato-protective	Monoterpenes	[111]
Cardio-protective	Monoterpenes	[95]
Gastro-protective	Monoterpenes	[95]
	-	[112]
	-	[113]
Anti-cholesterol and anti-atherogenic	-	[114]
	-	[115]
Anti-diabetic and hypoglycemic	-	[116]
	-	[117]
Estrogenic	Monoterpenes	[118]
	-	[119]
	-	[120]
Anti-anxiety	Monoterpenes	[121]
	Monoterpenes	[122]
	Monoterpenes	[123]
Nootropic	-	[124]

## Data Availability

No new data were created or analyzed in this study.

## References

[1] Chiocchio I., Mandrone M., Tomasi P., Marincich L., Poli F. (2021). Plant secondary metabolites: An opportunity for circular economy. Molecules.

[2] Giacometti J., Kovačević D.B., Putnik P., Gabrić D., Bilušić T., Krešić G., Stulić V., Barba F.J., Chemat F., Barbosa-Cánovas G. (2018). Extraction of bioactive compounds and essential oils from Mediterranean herbs by conventional and green innovative techniques: A review. Int. Food Res..

[3] Kabera J.N., Semana E., Mussa A.R., He X. (2014). Plant secondary metabolites: Biosynthesis, classification, function and pharmacological properties. J. Pharm. Pharmacol..

[4] Bourgaud F., Gravot A., Milesi S., Gontier E. (2001). Production of plant secondary metabolites: A historical perspective. Plant Sci..

[5] Demain A.L., Fang A., Fiechter A. (2000). The natural functions of secondary metabolites. History of Modern Biotechnology I, Advances in Biochemical Engineering/Biotechnology.

[6] Abbas M., Saeed F., Anjum F.M., Afzaal M., Tufail T., Bashir M.S., Ishtiaq A., Hussain S., Suleria H.A.R. (2017). Natural polyphenols: An overview. Int. J. Food Prop..

[7] Rasouli H., Farzaei M.H., Khodarahmi R. (2017). Polyphenols and their benefits: A review. Int. J. Food Prop..

[8] Sharma A., Shahzad B., Rehman A., Bhardwaj R., Landi M., Zheng B. (2019). Response of phenylpropanoid pathway and the role of polyphenols in plants under abiotic stress. Molecules.

[9] Radice M., Manfredini S., Ziosi P., Dissette V., Buso P., Fallacara A., Vertuani S. (2016). Herbal extracts, lichens and biomolecules as natural photo-protection alternatives to synthetic UV filters. A systematic review. Fitoterapia.

[10] Zillich O.V., Schweiggert-Weisz U., Eisner P., Kerscher M. (2015). Polyphenols as active ingredients for cosmetic products. Int. J. Cosmet. Sci..

[11] da Silva A.P.G., Sganzerla W.G., John O.D., Marchiosi R. (2023). A comprehensive review of the classification, sources, biosynthesis, and biological properties of hydroxybenzoic and hydroxycinnamic acids. Phytochem. Rev..

[12] Kumar N., Goel N. (2019). Phenolic acids: Natural versatile molecules with promising therapeutic applications. Biotechnol. Rep..

[13] Falcone Ferreyra M.L., Rius S.P., Casati P. (2012). Flavonoids: Biosynthesis, biological functions, and biotechnological applications. Front. Plant Sci..

[14] Das A.K., Islam M.N., Faruk M.O., Ashaduzzaman M., Dungani R. (2020). Review on tannins: Extraction processes, applications and possibilities. S. Afr. J. Bot..

[15] Xavier V., Spréa R., Finimundy T.C., Heleno S.A., Amaral J.S., Barros L., Ferreira I.C., Carocho M., Heleno S.A., Barros L. (2023). Terpenes. Natural Secondary Metabolites: From Nature, through Science, to Industry.

[16] Guimarães A.G., Serafini M.R., Quintans-Junior L.J. (2014). Terpenes and derivatives as a new perspective for pain treatment: A patent review. Expert Opin. Ther. Pat..

[17] Li C., Zha W., Li W., Wang J., You A. (2023). Advances in the biosynthesis of terpenoids and their ecological functions in plant resistance. Int. J. Mol. Sci..

[18] Holopainen J.K., Himanen S.J., Yuan J.S., Chen F., Stewart C.N., Ramawat K.G., Merillon J.M. (2013). Ecological functions of terpenoids in changing climates. Natural Products Phytochemistry, Botany and Metabolism of Alkaloids, Phenolics and Terpenes.

[19] Yang W., Chen X., Li Y., Guo S., Wang Z., Yu X. (2020). Advances in pharmacological activities of terpenoids. Nat. Prod. Commun..

[20] Barbulova A., Colucci G., Apone F. (2015). New trends in cosmetics: By-products of plant origin and their potential use as cosmetic active ingredients. Cosmetics.

[21] Aniszewski T. (2015). Alkaloids: Chemistry, Biology, Ecology, and Applications.

[22] Debnath B., Singh W.S., Das M., Goswami S., Singh M.K., Maiti D., Manna K. (2018). Role of plant alkaloids on human health: A review of biological activities. Mater. Today Chem..

[23] Ali A.H., Abdelrahman M., El-Sayed M.A., Jogaiah S., Abdelrahman M. (2019). Alkaloid role in plant defense response to growth and stress. Bioactive Molecules in Plant Defense.

[24] Garcia-Oliveira P., Barral M., Carpena M., Gullón P., Fraga-Corral M., Otero P., Prieto M.A., Simal-Gandara J. (2021). Traditional plants from Asteraceae family as potential candidates for functional food industry. Food Funct..

[25] Thiviya P., Gamage A., Piumali D., Merah O., Madhujith T. (2021). Apiaceae as an important source of antioxidants and their applications. Cosmetics.

[26] Sayed-Ahmad B., Talou T., Saad Z., Hijazi A., Merah O. (2017). The Apiaceae: Ethnomedicinal family as source for industrial uses. Ind. Crops Prod..

[27] Bessada S.M., Barreira J.C., Oliveira M.B.P. (2015). Asteraceae species with most prominent bioactivity and their potential applications: A review. Ind. Crops Prod..

[28] Milosavljevic S., Bulatovic V., Stefanovic M. (1999). Sesquiterpene lactones from the Yugoslavian wild growing plant families Asteraceae and Apiaceae. J. Serb. Chem. Soc..

[29] Holub M., Toman J., Herout V. (1987). The phylogenetic relationships of the Asteraceae and Apiaceae based on phytochemical characters. Biochem. Syst. Ecol..

[30] Chadwick M., Trewin H., Gawthrop F., Wagstaff C. (2013). Sesquiterpenoids lactones: Benefits to plants and people. Int. J. Mol. Sci..

[31] Denisow-Pietrzyk M., Pietrzyk L., Denisow B. (2019). Asteraceae species as potential environmental factors of allergy. Environ. Sci. Pollut. Res..

[32] Simonsen H.T., Weitzel C., Christensen S.B., Ramawat K.G., Merillon J.M. (2013). Guaianolide sesquiterpenoids: Pharmacology and biosynthesis. Natural Products.

[33] Chen L., Lu X., El-Seedi H., Teng H. (2019). Recent advances in the development of sesquiterpenoids in the treatment of type 2 diabetes. Trends Food Sci. Technol..

[34] Faustino M.V., Seca A.M., Silveira P., Silva A.M., Pinto D.C. (2017). Gas chromatography-mass spectrometry profile of four *Calendula* L. taxa: A comparative analysis. Ind. Crops Prod..

[35] Chitrakar B., Zhang M., Bhandari B. (2019). Edible flowers with the common name “marigold”: Their therapeutic values and processing. Trends Food Sci. Technol..

[36] Moghaddasi Mohammad S., Kashanisup H.H. (2012). Pot marigold (*Calendula officinalis*) medicinal usage and cultivation. Sci. Res. Essays.

[37] Arora D., Rani A., Sharma A. (2013). A review on phytochemistry and ethnopharmacological aspects of genus *Calendula*. Phcog. Rev..

[38] Olennikov D.N., Kashchenko N.I. (2022). Marigold metabolites: Diversity and separation methods of *Calendula* genus phytochemicals from 1891 to 2022. Molecules.

[39] Rather M.A., Dar B.A., Sofi S.N., Bhat B.A., Qurishi M.A. (2016). *Foeniculum vulgare*: A comprehensive review of its traditional use, phytochemistry, pharmacology, and safety. Arab. J. Chem..

[40] Badgujar S.B., Patel V.V., Bandivdekar A.H. (2014). *Foeniculum vulgare* Mill: A review of its botany, phytochemistry, pharmacology, contemporary application, and toxicology. Biomed Res. Int..

[41] Malhotra S.K., Peter K.V. (2012). Fennel and fennel seed. Handbook of Herbs and Spices.

[42] Wilson L., Caballero B., Finglas P.M., Toldrá F. (2016). Spices and flavoring crops: Fruits and seeds. Encyclopedia of Food and Health.

[43] Siviero P., Esposito C., De Masi L. (2005). Il finocchio (*Foeniculum vulgare* var. *dulce* Mill.). Ess. Der. Agr..

[44] Ashwlayan V.D., Kumar A., Verma M. (2018). Therapeutic potential of *Calendula officinalis*. Pharm. Pharmacol. Int. J..

[45] Shahane K., Kshirsagar M., Tambe S., Jain D., Rout S., Ferreira M.K.M., Mali S., Amin P., Srivastav P.P., Cruz J. (2023). An updated review on the multifaceted therapeutic potential of *Calendula officinalis* L.. Pharmaceuticals.

[46] Meikle R.D., Tutin T.G., Heywood V.H., Burges N.A., Valentine D.H., Walters S.M., Webb D.A. (1976). Calendula L. in Flora Europaea, 1.

[47] Safdar W., Majeed H., Naveed I., Kayani W.K., Ahmed H., Hussain S., Kamal A. (2010). Pharmacognostical study of the medicinal plant *Calendula officinalis* L. (family Compositae). Int. J. Cell Mol. Biol..

[48] de Lima Franzen F., Rodríguez de Oliveira M.S., Lidório H.F., Farias Menegaes J., Martins Fries L.L. (2019). Chemical composition of rose, sunflower and calendula flower petals for human food use. Cienc. Tecnol. Agropecu..

[49] Sapkota B., Kunwar P. (2024). A Review on Traditional Uses, Phytochemistry and Pharmacological Activities of Calendula officinalis Linn. Nat. Prod. Commun..

[50] Swiatek L., Gora J. (1978). Phenolic acids in the inflorescences of *Arnica montana* L. and *Calendula officinalis* L.. Herba Pol..

[51] Ak G., Zengin G., Sinan K.I., Mahomoodally M.F., Picot-Allain M.C.N., Cakır O., Bensari S., Yılmaz M.A., Gallo M., Montesano D. (2020). A comparative bio-evaluation and chemical profiles of *Calendula officinalis* L. extracts prepared via different extraction techniques. Appl. Sci..

[52] Ukiya M., Akihisa T., Yasukawa K., Tokuda H., Suzuki T., Kimura Y. (2006). Anti-inflammatory, anti-tumour-promoting, and cytotoxic activities of constituents of marigold (*Calendula officinalis*) flowers. J. Nat. Prod..

[53] Tavallali V., Rahmati S., Bahmanzadegan A., Lasibi M.J.M. (2024). Phenolic profile and evaluation of antimicrobial and anticancer activities of *Calendula officinalis* L. using exogenous polyamines application. Ind. Crops Prod..

[54] Yoshikawa M., Murakami T., Kishi A., Kageura T., Matsuda H. (2001). Medicinal flowers. III. Marigold. (1): Hypoglycemic, gastric emptying inhibitory, and gastroprotective principles and new oleanane-type triterpene oligoglycosides, calendasaponins A, B, C, and D, from Egyptian *Calendula officinalis*. Chem. Pharm. Bull..

[55] Olennikov D.N., Kashchenko N.I. (2013). New isorhamnetin glycosides and other phenolic compounds from *Calendula officinalis*. Chem. Nat. Compd..

[56] Okoh O.O., Sadimenko A.A., Afolayan A.J. (2007). The effects of age on the yield and composition of the essential oils of *Calendula officinalis*. J. Appl. Sci..

[57] Ak G., Zengin G., Ceylan R., Fawzi Mahomoodally M., Jugreet S., Mollica A., Stefanucci A. (2021). Chemical composition and biological activities of essential oils from *Calendula officinalis* L. flowers and leaves. Flavour Fragr. J..

[58] Adler G., Kasprzyk Z. (1975). Free sterols, steryl esters, glycosides, acelyted glycosides and water soluble complexes in *Calendula officinalis*. Phytochemistry.

[59] Wilkomirski B. (1985). Pentacyclic triterpene triols from *Calendula officinalis* flowers. Phytochemistry.

[60] Zitterl-Eglseer K., Reznicek G., Jurenitsch J., Novak J., Zitterl W., Franz C. (2001). Morphogenetic variability of faradiol monoesters in marigold *Calendula officinalis* L.. Phytochem. Anal..

[61] Sliwowski J., Dziewanowska K., Kasprzyk E. (1973). Ursadiol: A new triterpene diol from *Calendula officinalis* flowers. Khimija Prir. Soyedineniy.

[62] Wojciechowski Z., Bochenska-Hryniewicz M., Kurcharezak B., Kasprzyk Z. (1972). Sterol and triterpene alcohol esters from *Calendula officinalis*. Phytochemistry.

[63] Neukirch H., D’Ambrosio M., Via J.D., Guerriero A. (2004). Simultaneous quantitative determination of eight triterpenoid monoesters from flowers of 10 varieties of *Calendula officinalis* L. and characterisation of a new triterpenoid monoester. Phytochem. Anal..

[64] Bakò E., Deli J., Toth G. (2002). HPLC study on the carotenoid composition of calendula products. J. Biochem. Biophys. Methods.

[65] Kishimoto S., Maoka T., Sumitomo K., Ohmiya A. (2005). Analysis of carotenoid composition in petals of calendula (*Calendula officinalis* L.). Biosci. Biotechnol. Biochem..

[66] Hernández-Saavedra D., Pérez-Ramírez I.F., Ramos-Gómez M., Mendoza-Díaz S., Loarca-Pina G., Reynoso-Camacho R. (2016). Phytochemical characterization and effect of *Calendula officinalis*, *Hypericum perforatum*, and *Salvia officinalis* infusions on obesity-associated cardiovascular risk. Med. Chem. Res..

[67] Martinez L.G. (2020). Preclinical vascular activity of an aqueous extract from flowers of *Calendula officinalis*. J. Pharm. Pharmacol..

[68] Ray D., Mukherjee S., Falchi M., Bertelli A., Braga P.C., Das K.D. (2010). Amelioration of myocardial ischemic reperfusion injury with *Calendula officinalis*. Curr. Pharm. Biotechnol..

[69] Rigane G., Younes S.B., Ghazghazi H., Salem R.B. (2013). Investigation into the biological activities and chemical composition of *Calendula officinalis* L. growing in Tunisia. Int. Food Res. J..

[70] Frankic T., Salobir K., Salobir J. (2008). The comparison of in vivo antigenotoxic and antioxidative capacity of two propylene glycol extracts of *Calendula officinalis* (marigold) and vitamin E in young growing pigs. J. Anim. Physiol. Anim. Nutr..

[71] Braga P.C., Dal Sasso M., Culici M., Spallino A., Falchi M., Bertelli A., Morelli R., Lo Scalzo R. (2009). Antioxidant activity of *Calendula officinalis* extract: Inhibitory effects on chemiluminescence of human neutrophil bursts and electron paramagnetic resonance spectroscopy. Pharmacology.

[72] Efstratiou E., Hussain A.I., Nigam P.S., Moore J.E., Ayub M.A., Rao J.R. (2012). Antimicrobial activity of *Calendula officinalis* petal extracts against fungi, as well as Gram-negative and Gram-positive clinical pathogens. Complement. Ther. Clin. Pract..

[73] Darekar D., Hate M. (2021). Phytochemical screening of *Calendula officinalis* (Linn.) using gas-chromatography-mass spectroscopy with potential antibacterial activity. J. Sci. Res..

[74] Elias R., De Meo M., Vidal-Ollivier E., Laget M., Balansard G., Dumenil G. (1990). Antimutagenic activity of some saponins isolated from *Calendula officinalis* L., *C. arvensis* L. and *Hedera helix* L.. Mutagenesis.

[75] Jimenez-Medina E., Garcia-Lora A., Paco L., Algarra I., Collado A., Garrido F. (2006). A new extract of the plant *Calendula officinalis* produces a dual in vitro effect: Cytotoxic antitumor activity and lymphocyte activation. BMC Cancer.

[76] Cruceriu D., Balacescu O., Rakosy E. (2018). *Calendula officinalis*: Potential roles in cancer treatment and palliative care. Integr. Cancer Ther..

[77] Ercetin T., Senol F.S., Orhan I.E., Toker G. (2012). Comparative assessment of antioxidant and cholinesterase inhibitory properties of the marigold extracts from *Calendula arvensis* L. and *Calendula officinalis* L.. Ind. Crops Prod..

[78] Silva D., Ferreira M.S., Sousa-Lobo J.M., Cruz M.T., Almeida I.F. (2021). Anti-inflammatory activity of *Calendula officinalis* L. flower extract. Cosmetics.

[79] Nicolaus C., Junghanns S., Hartmann A., Murillo R., Ganzera M., Merfort I. (2017). In vitro studies to evaluate the wound healing properties of *Calendula officinalis* extracts. J. Ethnopharmacol..

[80] Preethi K.C., Kuttan R. (2009). Hepato and reno protective action of *Calendula officinalis* L. flower extract. Indian J. Exp. Biol..

[81] Bogdanova N.S., Nikolaeva I.S., Shcherbakova L.I., Tolstova T.I., Moskalenko N.I., Pershin G.N. (1970). Study of antiviral properties of *Calendula officinalis*. Farmakol. Toksikol..

[82] Kalvatchev Z., Walder R., Garzaro D. (1997). Anti-HIV activity of extracts from *Calendula officinalis* flowers. Biomed. Pharmacother..

[83] Reider N., Comericki P., Hausen B.M., Fritsch P., Aberer W. (2001). The seamy side of natural medicines: Contact sensitization to arnica (*Arnica montana* L.) and marigold (*Calendula officinalis* L.). Contact Dermat..

[84] Salapovic H., Geier J., Reznicek G. (2013). Quantification of sesquiterpene lactones in Asteraceae plant extracts: Evaluation of their allergenic potential. Sci. Pharm..

[85] Kooti W., Moradi M., Ali-Akbari S., Sharafi-Ahvazi N., Asadi-Samani M., Ashtary-Larky D. (2015). Therapeutic and pharmacological potential of *Foeniculum vulgare* Mill: A review. J. Herbmed Pharmacol..

[86] Diaz-Maroto M.C., Hidalgo I.J., Sanchez-Palomo E., Perez-Coello M.S. (2005). Volatile components and key odorants of fennel (*Foeniculum vulgare* Mill.) and thyme (*Thymus vulgaris* L.) oil extracts obtained by simultaneous distillation—Extraction and supercritical fluid extraction. J. Agric. Food Chem..

[87] Faudale M., Viladomat F., Bastida J., Poli F., Codina C. (2008). Antioxidant activity and phenolic composition of wild, edible, and medicinal fennel from different Mediterranean countries. J. Agric. Food Chem..

[88] Roby M.H.H., Sarhan M.A., Selim K.A., Khalel K.I. (2013). Antioxidant and antimicrobial activities of essential oil and extracts of fennel (*Foeniculum vulgare* L.) and chamomile (*Matricaria chamomilla* L.). Ind. Crops Prod..

[89] Parejo I., Jauregui O., Sánchez-Rabaneda F., Viladomat F., Bastida J., Codina C. (2004). Separation and characterization of phenolic compounds in fennel (*Foeniculum vulgare*) using liquid chromatography-negative electrospray ionization tandem mass spectrometry. J. Agric. Food Chem..

[90] Marino S.D., Gala F., Borbone N., Zollo F., Vitalini S., Visioli F., Iorizzi M. (2007). Phenolic glycosides from *Foeniculum vulgare* fruit and evaluation of antioxidative activity. Phytochemistry.

[91] Diaz-Maroto M.C., Perez-Coello M.S., Esteban J., Sanz J. (2006). Comparison of the volatile composition of wild fennel samples (*Foeniculum vulgare* Mill.) from Central Spain. J. Agric. Food Chem..

[92] Damjanovic B., Lepojevic Z., Zivkovic V., Tolic A. (2005). Extraction of fennel (*Foeniculum vulgare* Mill.) seeds with supercritical CO2: Comparison with hydrodistillation. Food Chem..

[93] Vella F.M., Calandrelli R., Cautela D., Fiume I., Pocsfalvi G., Laratta B. (2020). Chemometric screening of fourteen essential oils for their composition and biological properties. Molecules.

[94] Cautela D., Vella F.M., Castaldo D., Laratta B. (2019). Characterization of essential oil recovered from fennel horticultural wastes. Nat. Prod. Res..

[95] Tognolini M., Ballabeni V., Bertoni S., Bruni R., Impicciatore M., Barocelli E. (2007). Protective effect of Foeniculum vulgare essential oil and anethole in an experimental model of thrombosis. Pharmacol. Res..

[96] Senatore F., Oliviero F., Scandolera E., Taglialatela-Scafati O., Roscigno G., Zaccardelli M., De Falco E. (2013). Chemical composition, antimicrobial and antioxidant activities of anethole-rich oil from leaves of selected varieties of fennel [*Foeniculum vulgare* Mill. ssp. *vulgare* var. *azoricum* (Mill.) Thell]. Fitoterapia.

[97] Diao W., Hu Q., Zhang H., Xu J. (2014). Chemical composition, antibacterial activity and mechanism of action of essential oil from seeds of fennel (*Foeniculum vulgare* Mill.). Food Control.

[98] Shahat A.A., Ibrahim A.Y., Hendawy S.F., Omer E.A., Hammouda F.M., Rahman F.H.A., Saleh M.A. (2011). Chemical composition, antimicrobial and antioxidant activities of essential oils from organically cultivated fennel cultivars. Molecules.

[99] Servi H., Şen A., Yildirim S., Doğan A. (2021). Chemical composition and biological activities of essential oils of *Foeniculum vulgare* Mill. and *Daucus carota* L. growing wild in Turkey. J. Res. Pharm..

[100] Afifi S.M., El-Mahis A., Heiss A.G., Farag M.A. (2021). Gas chromatography-mass spectrometry-based classification of 12 fennel (*Foeniculum vulgare* Miller) varieties based on their aroma profiles and estragole levels as analyzed using chemometric tools. ACS Omega.

[101] Kaur G.J., Arora D.S. (2009). Antibacterial and phytochemical screening of *Anethum graveolens*, *Foeniculum vulgare* and *Trachyspermum ammi*. BMC Complement. Med. Ther..

[102] Schulz M., Meins J., Diemert S., Zagermann-Muncke P., Goebel R., Schrenk D., Schubert-Zsilavecz M., Abdel-Tawab M. (2015). Detection of pyrrolizidine alkaloids in German licensed herbal medicinal teas. Phytomedicine.

[103] Kwon Y., Koo Y., Jeong Y. (2021). Determination of pyrrolizidine alkaloids in teas using liquid chromatography-tandem mass spectrometry combined with rapid-easy extraction. Foods.

[104] Zellagui A., Gherraf N., Elkhateeb A., Hegazy M.E.F., Mohamed T.A., Touil A., Shahat A.A., Rhouati S. (2011). Chemical constituents from Algerian *Foeniculum vulgare* aerial parts and evaluation of antimicrobial activity. J. Chil. Chem. Soc..

[105] Dadalioglu I., Evrendilek G.A. (2004). Chemical compositions and antibacterial effects of essential oils of turkish oregano (*Origanum minutiflorum*), bay laurel (*Laurus nobilis*), spanish lavender (*Lavandula stoechas* L.), and fennel (*Foeniculum vulgare*) on common foodborne pathogens. J. Agric. Food Chem..

[106] Cetin B., Ozer H., Cakir A., Polat T., Dursun A., Mete E., Oztürk E., Ekinci M. (2010). Antimicrobial activities of essential oil and hexane extract of Florence fennel [*Foeniculum vulgare* var. *azoricum* (Mill.) Thell.] against foodborne microorganisms. J. Med. Food.

[107] Orhan I.E., Ozcelik B., Kartal M., Kan Y. (2012). Antimicrobial and antiviral effects of essential oils from selected Umbelliferae and Labiatae plants and individual essential oil components. Turk. J. Biol..

[108] Chainy G.B., Manna S.K., Chaturvedi M.M., Aggarwal B.B. (2000). Anethole blocks both early and late cellular responses transduced by tumor necrosis factor: Effect on NF-κB, AP-1, JNK, MAPKK and apoptosis. Oncogene.

[109] Pradhan M., Sribhuwaneswari S., Karthikeyan D., Minz S., Sure P., Chandu A.N., Mishra U., Kamalakannan K., Saravanankumar A., Sivakumar T. (2008). In vitro cytoprotection activity of *Foeniculum vulgare* and *Helicteres isora* in cultured human blood lymphocytes and antitumor activity against B16F10 melanoma cell line. Res. J. Pharm. Technol..

[110] Mohamad R.H., El-Bastawesy A.M., Abdel-Monem M.G., Noor A.M., Al-Mehdar H.A.R., Sharawy S.M., El-Merzabani M.M. (2011). Antioxidant and anticarcinogenic effects of methanolic extract and volatile oil of fennel seeds (*Foeniculum vulgare*). J. Med. Food.

[111] Ozbek H., Ugraş S., Dulger H., Bayram I., Tuncer I., Ozturk G., Ozturk A. (2003). Hepatoprotective effect of *Foeniculum vulgare* essential oil. Fitoterapia.

[112] Birdane F.M., Cemek M., Birdane Y.O., Gülçin I., Büyükokuroğlu M.E. (2007). Beneficial effects of Foeniculum vulgare on ethanol-induced acute gastric mucosal injury in rats. World J. Gastroenterol..

[113] Al-Mofleh I., Al-Sobaihani M., Alqasoumi S., Al-Said M., Al-Dosari M., Al-Yahya M., Rafatullah S. (2013). Fennel *Foeniculum vulgare* treatment protects the gastric mucosa of rats against chemically-induced histological lesions. Int. J. Pharm..

[114] Oulmouden F., Ghalim N., El Morhit M., Benomar H., Daoudi E.M., Amrani S. (2014). Hypolipidemic and anti-atherogenic effect of methanol extract of fennel (*Foeniculum vulgare*) in hypercholesterolemic mice. Int. J. Sci. Knowl..

[115] Sushruta K., Satyanarayana S., Srinivas S., Sekhar J.R. (2007). Evaluation of the blood-glucose reducing effects of aqueous extracts of the selected umbelliferous fruits used in culinary practices. Trop. J. Pharm. Res..

[116] Dongare V., Arvindekar A., Magadum C. (2010). Hypoglycemic effect of *Foeniculum vulgare* Mill. fruit on dexamethasone induced insulin resistance rats. Res. J. Pharmacogn. Phytochem..

[117] El-Soud N., El-Laithy N., El-Saeed G., Wahby M., Khalil M., Morsy F., Shaffie N. (2011). Antidiabetic activities of *Foeniculum vulgare* Mill. essential oil in streptozotocin-induced diabetic rats. Maced. J. Med. Sci..

[118] Albert-Puleo M. (1980). Fennel and anise as estrogenic agents. J. Ethnopharmacol..

[119] Ostad S., Soodi M., Shariffzadeh M., Khorshidi N., Marzban H. (2011). The effect of fennel essential oil on uterine contraction as a model for dysmenorrhea, pharmacology and toxicology study. J. Ethnopharmacol..

[120] Myrseyed F., Shiravi A., Nasr-Abadi M. (2008). The effect of intraperitoneal injection of alcoholic extract *Foeniculum vulgare* seed on gonadotropic and testosterone hormones in male wistar rats. J. Anim. Sci..

[121] Mesfin M., Asres K., Shibeshi W. (2014). Evaluation of anxiolytic activity of the essential oil of the aerial part of *Foeniculum vulgare* Miller in mice. BMC Complement. Altern. Med..

[122] Pourabbas S., Kesmati M., Rasekh A. (2011). Study of the anxiolytic effects of fennel and possible roles of both gabaergic system and estrogen receptors in these effects in adult female rat. Physiol. Pharmacol..

[123] Lima N.G., De Sousa D.P., Pimenta F.C.F., Alves M.F., De Souza F.S., Macedo R.O., Cardoso R.B., de Morais L.C., De Almeida R.N. (2013). Anxiolytic-like activity and GC-MS analysis of (R)-(+)-limonene fragrance, a natural compound found in foods and plants. Pharmacol. Biochem. Behav..

[124] Joshi H., Parle M. (2006). Cholinergic basis of memory-strengthening effect of *Foeniculum vulgare* Linn. J. Med. Food.

[125] Zeller A., Rychlik M. (2006). Character impact odorants of fennel fruits and fennel tea. J. Agric. Food Chem..

[126] Drinkwater N.R., Miller E.C., Miller J.A., Pitot H.C. (1976). Hepato-carcinogenicity of estragole (1-allyl-4-methoxybenzene) and 1-hydroxyestragole in the mouse and mutagenicity of 1-acetoxyestragole in bacteria. J. Natl. Cancer Inst..

[127] Riboli E., Beland F.A., Lachenmeier D.W., Marques M.M., Phillips D.H., Schernhammer E., Afghan A., Assunção R., Caderni G., Corton J.C. (2023). Carcinogenicity of aspartame, methyleugenol, and isoeugenol. Lancet Oncol..

[128] Paini A., Punt A., Viton F., Scholz G., Delatour T., Marin-Kuan M., Schilter B., van Bladeren P.J., Rietjens I.M. (2010). A physiologically based biodynamic (PBBD) model for estragole DNA binding in rat liver based on in vitro kinetic data and estragole DNA adduct formation in primary hepatocytes. Toxicol. Appl. Pharmacol..

[129] Miller E.C., Swanson A.B., Phillips D.H., Fletcher T.L., Liem A., Miller J.A. (1983). Structure-activity studies of the carcinogenicities in the mouse and rat of some naturally occurring and synthetic alkenyl-benzene derivatives related to safrole and estragole. Cancer Res..

[130] Swanson A.B., Miller E.C., Miller J.A. (1981). The side-chain epoxidation and hydroxylation of the hepatocarcinogens safrole and estragole and some related compounds by rat and mouse liver microsomes. Biochim. Biophys. Acta.

[131] Swanson A.B., Chambliss D.D., Blomquist J.C., Miller E.C., Miller J.A. (1979). The mutagenicities of safrole, estragole, eugenol, trans-anethole, and some of their known or possible metabolites for *Salmonella typhimurium* mutants. Mutat. Res..

[132] Punt A., Freidig A.P., Delatour T., Scholz G., Boersma M.G., Schilter B., van Bladeren P.J., Rietjens I.M. (2008). A physiologically based biokinetic (PBBK) model for estragole bioactivation and detoxification in rat. Toxicol. Appl. Pharmacol..

